# Effects of Sodium–Glucose Cotransporter 2 Inhibitors on Transcription Regulation of *AgRP* and *POMC* Genes

**DOI:** 10.3390/cimb46070445

**Published:** 2024-07-15

**Authors:** Dong Hee Kim, Min Jin Lee, Dasol Kang, Ah Reum Khang, Ji Hyun Bae, Joo Yeon Kim, Su Hyun Kim, Yang Ho Kang, Dongwon Yi

**Affiliations:** 1 Department of BIT Fusion Technology Center, Pusan National University, Busan 46241, Republic of Korea; gainlydh@pusan.ac.kr; 2 Research Institute for Convergence of Biomedical Science and Technology, Pusan National University Yangsan Hospital, Yangsan 50612, Republic of Korea; minjinpig@hanmail.net (M.J.L.); medikar82@gmail.com (A.R.K.); yaho1229@gmail.com (J.H.B.); rlawndus0226@naver.com (J.Y.K.); carpediem1113@gmail.com (S.H.K.); kangyh@pusan.ac.kr (Y.H.K.); 3 Division of Endocrinology and Metabolism, Department of Internal Medicine, Pusan National University Yangsan Hospital, Pusan National University School of Medicine, Yangsan 50612, Republic of Korea; 4 Department of Biological Sciences, College of National Sciences, University of Ulsan, Ulsan 44919, Republic of Korea; lainef7@ulsan.ac.kr

**Keywords:** SGLT2 inhibitors, energy homeostasis, hypothalamus

## Abstract

Sodium–glucose cotransporter 2 (SGLT2) inhibitors regulate plasma glucose levels in patients with type 2 diabetes mellitus (T2DM) by inhibiting renal glucose reabsorption. This study investigated the impact of empagliflozin (EMPA), an SGLT2 inhibitor, on hypothalamic energy regulation. To directly investigate the role of SGLT2 inhibitors in the hypothalamus, we administered EMPA through intracerebroventricular (i.c.v.) injections into the murine ventricles. After dental cementing the i.c.v. cannula onto the skull, the mice were given 5 days to recover before receiving vehicle or EMPA (50 nM/2 μL) injections. In a high-fat diet (HFD)-induced obesity model, we determined the gene expression levels of agouti-related peptide (*AgRP*) and pro-opiomelanocortin (*POMC*) in the hypothalamus. Additionally, we assessed FoxO1 expression, which regulates *AgRP* and *POMC* gene transcription in hypothalamic cell lines. We found that EMPA directly influenced the expression of endogenous mRNA of POMC and AgRP, which are critical for energy homeostasis, and modulated their transcription in high-fat diet-induced obese mice. Additionally, EMPA affected the expression of FoxO1, a key transcriptional regulator of glucose homeostasis, thereby regulating the transcriptional activity of *POMC* and *AgRP*. These results indicate that EMPA significantly influences hypothalamic energy homeostasis, highlighting its potential as a regulator in obesity and T2DM management.

## 1. Introduction

There is a strong interrelation between type 2 diabetes mellitus (T2DM) and obesity, sharing pathophysiological mechanisms and leading to severe long-term complications [1]. Obesity can trigger several physiological issues, including hypertension [2], dyslipidemia [3], and insulin resistance [4,5], which in turn elevate the risk of developing T2DM. Obesity-induced inflammation exacerbates insulin resistance and disrupts glycemic control. Therefore, understanding the link between obesity and T2DM is critical [6,7], particularly as obesity-associated T2DM is characterized by impaired glucose homeostasis. While the pathogenesis of these metabolic disorders is not fully understood, “weight loss” is a critical aspect of managing diabetes mellitus. Effective appetite control is central to achieving weight loss.

The central nervous system (CNS) is crucial for continuously monitoring circulating glucose levels and coordinating with peripheral organs to control glucose levels [8,9]. In the CNS, the hypothalamus is the primary region involved in glucose regulation, containing various nuclei that produce neuropeptides and neurotransmitters essential for energy homeostasis. The arcuate nucleus (ARC) is crucial in detecting the body’s overall energy status. It regulates metabolic balance through two types of neurons: anorexigenic pro-opiomelanocortin (POMC) neurons, which decrease appetite and boost energy expenditure, and orexigenic neuropeptide Y/agouti-related peptide (NPY/AgRP) neurons, which promote food consumption and energy storage [10,11]. Further research has revealed that food intake regulation involves complex mechanisms, including contributions from the CNS, various hormones, and gastrointestinal polypeptides [12]. The control of appetite and energy expenditure via these mechanisms is intricately connected to the onset of obesity and T2DM.

Recently, sodium–glucose cotransporter 2 (SGLT2) inhibitors have been approved for managing T2DM by reducing blood glucose levels via an insulin-independent pathway [13]. These inhibitors promote the excretion of glucose in urine by blocking its reabsorption in the proximal tubule of the kidney [14]. Recent studies have demonstrated that SGLT2 inhibitors offer numerous benefits, including renal protection [15,16], reduced risk of cardiovascular diseases, lowered blood pressure [17,18], and weight loss [19,20].

SGLT2 inhibitors have demonstrated weight reduction effects in patients with T2DM and obese mice models. Previously, these effects were primarily attributed to caloric loss due to glucose excretion and weight loss due to fluid reduction via osmotic diuresis [21,22,23,24]. Recently, with the elucidation of the browning mechanism of white adipose tissue, these inhibitors are being proposed as a basis for obesity treatment [25,26]. Regulating eating behavior and other physiological functions through drug administration has been a long-term objective in clinical research. Recent research has investigated whether the advantageous effects of empagliflozin (EMPA), a member of SGLT2 inhibitors, may be partially mediated through specific CNS behaviors, although the precise mechanisms remain unclear [27]. Our current understanding of how SGLT2 inhibitors impact various appetite-regulating neuropeptides and weight control mechanisms remains limited. Therefore, in this study, we aimed to focus on weight control mechanisms by examining the patterns of appetite-regulating neuropeptides induced by SGLT2 inhibitors, which represent a central regulatory mechanism.

## 2. Materials and Methods

### 2.1. Animals

Eight-week-old male C57BL/6N mice, sourced from Koatech (Namyangju-si, Republic of Korea), were used for experimentation, with approval from the Institutional Animal Care and Use Committee at the Pusan National University Yangsan Hospital Biomedical Research Institute (LT2024-012-A1C0). The mice were housed in a controlled environment, with a 12 h light/dark cycle (lights on from 7:00 a.m. to 7:00 p.m.) and a temperature range of 23–25 °C. After being fed a standard diet (normal-fat diet [NFD], *n* = 4, containing 10% kcal fat diet) or high-fat diet (HFD, *n* = 5, containing 60% kcal fat diet) for 16 weeks, their food intake was recorded for 1 week. This was followed by 3 weeks of EMPA treatment (HFD + EMPA, *n* = 5 (once per week each for 3 weeks, EMPA at a dose of 10 mg/kg/day by oral gavage), during which food intake was also monitored. Food intake was assessed weekly by measuring the amount of remaining food in each cage. The average daily food consumption was calculated by dividing the total food consumed by the number of days in the measurement period and the number of mice per cage. Cumulative food intake was computed by adding the weekly food consumption per cage and dividing by the number of mice in the cage.

### 2.2. Cannulation and Administration of EMPA

Intracerebroventricular (i.c.v.) cannulation was performed as previously described [28]. After a recovery period of 5 days, the mice received either vehicle (*n* = 8) or EMPA (*n* = 11, 50 nM/2 μL [1 μL/min]) injections.

### 2.3. Cell Culture and Luciferase Assay

The mouse hypothalamic mHypoA 2/28 cells were grown as previously reported [29]. To investigate the impact of EMPA on *POMC* and *AgRP* transcriptional activity, cells were transfected with either a *POMC* promoter–luciferase reporter vector (500 ng) or an *AgRP* promoter–luciferase reporter vector (500 ng) using jetPRIME^®^ reagent (Polyplus, New York, NY, USA). To normalize transfection efficiency, a Renilla reporter plasmid (pRL-SV40 vector; Promega, Madison, WI, USA) was co-transfected at 100 ng/well. The transfected cells were collected 24 h post-transfection, and luciferase activity was quantified using a luciferase reporter assay system (Promega) as per the manufacturer’s instructions.

### 2.4. RNA Isolation and Quantitative Real-Time Polymerase Chain Reaction (PCR)

Total RNA was extracted from the hypothalamus and hypothalamic cell lines using the Sensi-TriJol reagent (Lugen SCI, Inc., Bucheon, Republic of Korea) according to the manufacturer’s instructions. For cDNA synthesis, 2 μg of total RNA was reverse-transcribed using the UltraScript 2.0 cDNA Synthesis Kit (PCR Biosystems, London, England) and amplified by real-time PCR using the following primer sets. The primer sequences are as follows: AgRP sense primer, 5′-AAT GTT GCT GAG TTG TGT TCT G-3′; AgRP antisense primer, 5′-GGC CAT TCA GAC TTA GAC CTG-3′; POMC sense primer, 5′-GCC TTT CCC CTA GAG TTC AAG-3′; POMC antisense primer, 5′-ACC GTA ACG CTT GTC CTT G-3′; FoxO1 sense primer, 5′-CAG ACA CTT CAG GAC AGC AA-3′; FoxO1 antisense primer, 5′-AAG CTG TGG CGT GAT GGC-3′; β-actin sense primer, 5′-GGC TGT ATT CCC CTC CAT CG-3′; β-actin antisense primer, 5′-CCA GTT GGT AAC AAT GCC ATG T-3′. The data were normalized for gene expression using β-actin as an internal control. Real-time PCR amplification of the cDNA was performed using the SYBR Green Real-time PCR Master Mix (Toyobo Co., Ltd., Osaka, Japan) on Light Cycler 480 (Roche Diagnostics Ltd., Rotkreuz, Switzerland) for ~40 cycles. Relative gene expressions were calculated using the 2^−△△CT^ method [30].

### 2.5. Cell Viability Assay

The viability of mHypoA cells was assessed according to the method previously described by van Meerloo [31]. Absorbance at 540 nm was measured using a microplate spectrophotometer (BioTek Instruments, Winooski, VT, USA).

### 2.6. Small Interfering RNA (siRNA) Transfection

For knocking down the endogenous synthesis of FoxO1 in hypothalamic cells, a siRNA was constructed by automated solid phase synthesis (Bioneer, Daejeon, Republic of Korea) and was transfected into the mHypoA cells using jetPRIME^®^ reagent. The FoxO1 siRNA sequence was designed using exon 3 of the FoxO1 cDNA sequence (NCBI GenBank accession number: NM_002015.3) as follows: 5′-CUG CAU AGC AUC AAG UCU U-3′ and 5′-AAG ACU UGA UGC UAU GCA G-3′. AccuTarget™ Negative Control siRNA (Bioneer) was used as a negative control.

### 2.7. Immunoblotting

Proteins from mHypoA cells were homogenized using an M-PER lysis buffer (Pierce, Waltham, MA, USA) containing a protease inhibitor cocktail (Roche, Basel, Switzerland). Extracted protein concentrations were quantitated using the Bradford assay (Bio-Rad Laboratories, Hercules, CA, USA) and were separated by sodium dodecyl sulfate–polyacrylamide gel electrophoresis and transferred to a polyvinylidene difluoride membrane (GE Healthcare, Piscataway, NJ, USA) and treated with either an anti-FoxO1 antibody (1:1000; Cell Signaling Technology, Beverly, MA, USA) or an anti-β-actin antibody (1:10,000; Sigma Aldrich). Immunoreactivity was detected using an enhanced chemiluminescence kit (Amersham Biosciences, Piscataway, NJ, USA).

### 2.8. Statistical Analyses

Statistical analyses were conducted using GraphPad Prism 10 software (GraphPad Software, San Diego, CA, USA). Data are presented as mean ± standard error of the mean. A one-way or two-way analysis of variance followed by Tukey’s multiple comparison test for unequal replications was used to analyze the data. Comparisons between the two groups were performed using Student’s *t*-test.

## 3. Results

### 3.1. Effect of Directly Administered EMPA on the Mouse Brain

**Figure 1:**
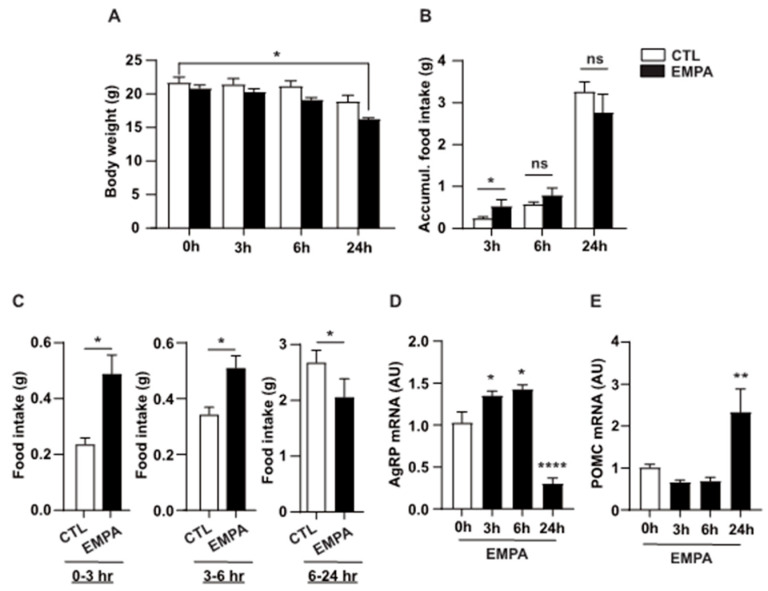
Effects of i.c.v. EMPA administration on body weight, food intake, and AgRP and POMC mRNA levels. Eight-week-old male mice were intracerebroventricularly injected with either vehicle or EMPA. (**A**) Body weight and (**B**,**C**) cumulative food intake are measured for 24 h post-injection. (**D**,**E**) Real-time PCR analysis reveals that AgRP mRNA increased during the first 6 h following EMPA injection but decreased significantly by 24 h. (**E**) POMC mRNA shows no immediate changes post-injection but increased by 24 h. Data are presented as mean ± SEM. ns, not significant; *, *p* < 0.05; **, *p* < 0.01; ****, *p* < 0.0001. Abbreviations: AgRP, agouti-related peptide; CTL, control; EMPA, empagliflozin; i.c.v., intracerebroventricular; PCR, polymerase chain reaction; POMC, pro-opiomelanocortin; SEM, standard error of the mean.

A prior study indicated that SGLT2 inhibitor administration influences appetite and weight regulation in both patients with diabetes and animal models [32]. To examine the direct impact of SGLT2 inhibitors on the brain, we injected EMPA directly into the lateral ventricle of 8-week-old male mice. We then investigated changes in AgRP and POMC mRNA levels in the hypothalamus of mice. Food intake and body weight changes were observed at three time-points (3, 6, and 24 h) after EMPA injection. Hypothalamus tissue samples were collected to assess changes in AgRP and POMC mRNA levels. A reduction in body weight was observed at 24 h after CNS administration of EMPA (Figure 1A). Subsequently, we assessed cumulative food intake, which was increased by EMPA at 3 h; however, food intake did not differ significantly between the control and EMPA groups at 6 and 24 h (Figure 1B). However, when analyzing food intake over time-points rather than cumulative intake, we noticed a trend of increased intake induced by EMPA until 24 h, followed by a decrease (Figure 1C). Consequently, brain samples were collected at each time-point to analyze the mRNA expression of *AgRP* and *POMC*, genes involved in appetite regulation. Consistent with the intake pattern, AgRP mRNA expression increased at 3 and 6 h after EMPA injection, while POMC mRNA expression significantly increased at 24 h (Figure 1D,E). These findings imply that EMPA might modulate the expression of genes related to appetite and energy metabolic regulation within the CNS, potentially affecting body weight changes.

### 3.2. Effect of EMPA in HFD-Induced Obese Mice

**Figure 2:**
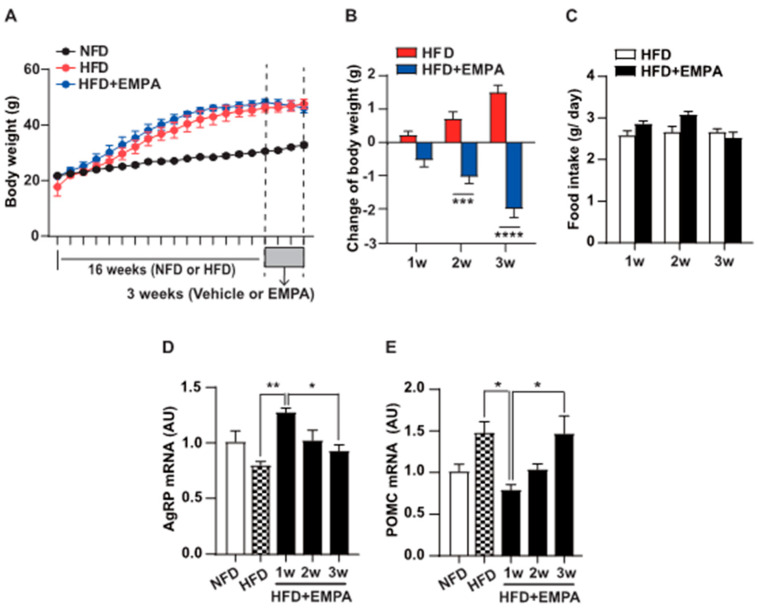
Effect of EMPA on variations in body weight, food intake, and AgRP and POMC mRNA levels. Body weight and food intake of mice treated with vehicle or EMPA were measured for 3 weeks after 16 weeks of standard diet (NFD) or high-fat diet (HFD) conditions. (**A**,**B**) Significant reductions in body weight are observed in mice treated with EMPA under high-fat diet conditions starting from the seco week. (**C**) While mice treated with EMPA show an increasing trend in food intake from the second week, it is observed to decrease in the third week instead. (**D**,**E**) Real-time PCR analysis reveals that AgRP mRNA increased during the first week after EMPA injection on a high-fat diet, while POMC mRNA significantly decreased. However, in mice injected with EMPA by the third week, AgRP mRNA decreased while POMC mRNA increased. Data are presented as mean ± SEM. *, *p* < 0.05; **, *p* < 0.01; ***, *p* < 0.001; ****, *p* < 0.0001. Abbreviations: AgRP, agouti-related peptide; EMPA, empagliflozin; PCR, polymerase chain reaction; POMC, pro-opiomelanocortin; SEM, standard error of the mean.

Next, we investigated the role of EMPA in obesity-induced metabolic syndrome using 8-week-old mice. These mice were fed either an HFD or a standard diet (normal fat diet [NFD]) for 16 weeks, followed by a 3-week treatment period where the treatment group received EMPA or an equivalent amount of vehicle. Body weight in the HFD group was significantly higher than that in the NFD group starting from the fourth week (Figure 2A), with the difference becoming more pronounced over time. Subsequently, treatment with EMPA for 3 weeks revealed a suppression of weight gain compared to the group treated with HFD alone (Figure 2B). We tracked food intake changes weekly after EMPA administration. While EMPA-treated mice exhibited increased food intake compared to the vehicle-treated group during the first and second weeks, a decrease was observed in the third week; the difference was not statistically significant (Figure 2C). After observing changes in body weight and feeding behavior, we examined the mRNA expression of AgRP (Figure 2D) and POMC (Figure 2E) in the hypothalamus. Unexpectedly, we found an increase in AgRP mRNA expression in the EMPA-treated group compared to the control group during the first week, which decreased over time. Furthermore, we discovered a significant increase in POMC mRNA expression 3 weeks after exposure to EMPA. These findings indicate that EMPA may have various physiological effects in obesity-induced metabolic syndrome.

### 3.3. Effect of EMPA on the Transcriptional Regulation of AgRP and POMC Genes in the Hypothalamic Cell Line

**Figure 3:**
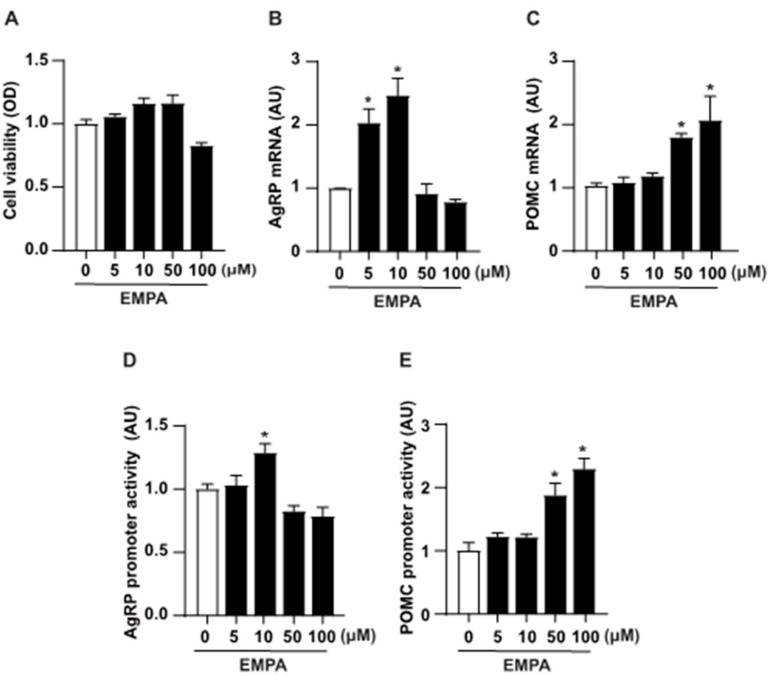
Effect of EMPA on the transcriptional regulation of the *AgRP* and *POMC* genes. (**A**) The viability of mHypoA cells is assessed using a 3-(4,5-dimethylthiazol-2-yl)-2,5-diphenyltetrazolium bromide assay. The cells are treated with varying concentrations of EMPA (0–100 μM) for 24 h in a dose-dependent manner. EMPA is treated into mHypoA cells, and cells were harvested for real-time PCR. Real-time PCR analysis reveals changes in (**B**) AgRP mRNA and (**C**) POMC mRNA. Luciferase reporter constructs (pGL3) containing the 5′-flanking region of the *AgRP* and *POMC* genes are transfected into mHypoA cells and treated with EMPA. (**D**) Transactivation of the *AgRP* promoter with low concentrations of EMPA. (**E**) Increased activity of the *POMC* promoter at high concentrations. Data are presented as mean ± SEM. The data represent the mean of at least three repeated measurements. * *p* < 0.05 vs. control. Abbreviations: AgRP, agouti-related peptide; EMPA, empagliflozin; PCR, polymerase chain reaction; POMC, pro-opiomelanocortin; SEM, standard error of mean.

We investigated the transcriptional regulation of *AgRP* and *POMC* by EMPA using the mouse hypothalamic mHypoA cell line. We evaluated the influence of EMPA, in a dose-dependent manner, on cell viability in mHypoA cells using MTT assays. Our results showed that even at high concentrations, EMPA minimally impacted cell viability (Figure 3A). Subsequently, we employed in vitro experiments with mHypoA cells to investigate the impact of EMPA on the expression of AgRP and POMC mRNA. The mHypoA cells were subjected to treatment with EMPA under dose-dependent conditions. Real-time PCR analysis was utilized to quantify the transcription levels of AgRP and POMC mRNA (Figure 3B,C). The gene expression pattern of AgRP and POMC mRNA elicited a dose-dependent response to EMPA. In particular, levels of AgRP mRNA expression increased at low concentrations, whereas POMC mRNA expression increased at high concentrations of EMPA. To validate EMPA’s regulatory effect on the transcription of the *AgRP* and *POMC* genes, promoter assays employed a luciferase construct containing the *AgRP* and *POMC* promoter regions in mHypoA cells. *AgRP* promoter activity gradually increased upon treatment with a specific dose (10 μM) of EMPA in mHypoA cells (Figure 3D), whereas *POMC* promoter activity was enhanced with high doses of EMPA treatment (Figure 3E). This suggests a dose-dependent modulation of key genes involved in hypothalamic signaling pathways by EMPA.

### 3.4. Effect of EMPA on AgRP and POMC Transcriptional Regulation via FoxO1 Activation

**Figure 4:**
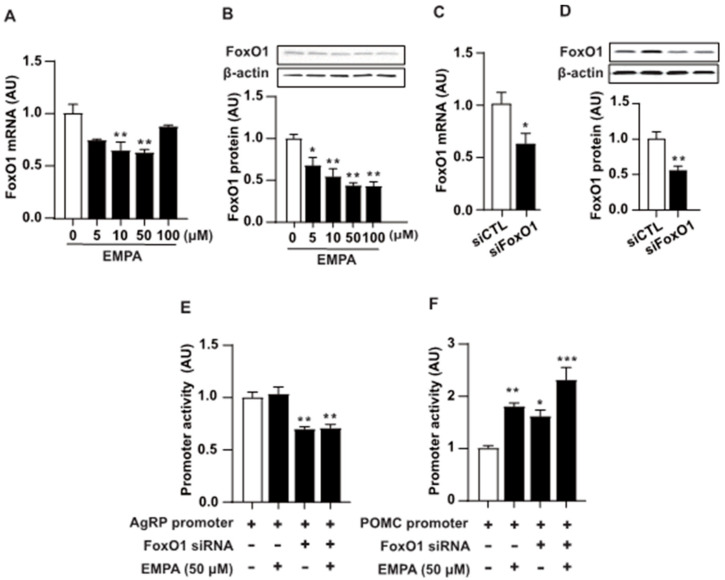
The transcriptional regulation of *AgRP* and *POMC* genes by FoxO1 under EMPA-treated conditions. EMPA’s effect on FoxO1 expression is determined using EMPA-treated mHypoA cells. Treatment with specified concentrations of EMPA results in a significant decrease in (**A**) FoxO1 mRNA and (**B**) protein levels as compared to the control. (**C**,**D**) Subsequently, we chose to use FoxO1 siRNA to verify the effect of FoxO1 reduction, and the efficiency of siRNA was confirmed. (**E**,**F**) The inhibitory and stimulatory effects of FoxO1 and EMPA treatment on *AgRP* and *POMC* promoter activities are examined. The results show a decrease in *AgRP* promoter activity due to FoxO1 inhibition, but no significant effect is observed with EMPA treatment. However, for *POMC*, both FoxO1 and EMPA demonstrate significant effects on promoter activity. Data are presented as mean ± SEM. The data represent the mean of at least three repeated measurements. *, *p* < 0.05; **, *p* < 0.01; ***, *p* < 0.001 vs. control. Abbreviations: AgRP, agouti-related peptide; EMPA, empagliflozin;; POMC, pro-opiomelanocortin; SEM, standard error of mean; siCTL, small interfering control; siFoxO1, small interfering FoxO1, siRNA, small interfering RNA.

Previous studies have reported that the SGLT2 inhibitor regulates the transcription factor FoxO1 [33,34]. FoxO1 plays a key role in appetite regulation and energy metabolism by modulating the expression of *AgRP* and *POMC* [35,36,37]. Therefore, we aimed to determine whether the regulatory effects of EMPA on *AgRP* and *POMC* are mediated through FoxO1. To determine EMPA action on the endogenous FoxO1 expression, we performed real-time PCR and immunoblotting analysis of mRNA and protein extract from the mHypoA cells treated with EMPA. EMPA decreased endogenous mRNA (Figure 4A) as well as protein (Figure 4B) levels of FoxO1. To assess the effects of EMPA and FoxO1 inhibition on the expression of *AgRP* and *POMC*, we utilized siRNA targeting FoxO1 in mHypoA cells (Figure 4C,D). Furthermore, to evaluate the specific effects of EMPA and FoxO1 inhibition on the expression of *AgRP* and *POMC*, we transfected mHypoA cells with FoxO1-targeting siRNA, followed by assessment of their activity using the *AgRP* and *POMC* promoters in response to EMPA (Figure 4E,F). As a result, the *POMC* promoter exhibited a significant increase in response to EMPA and FoxO1 inhibition (Figure 4E); however, no effect mediated by EMPA-induced FoxO1 inhibition was observed in AgRP activity (Figure 4F). These findings suggest that the decrease in the levels of FoxO1 induced by EMPA may be responsible for the increase in POMC transcriptional activity and indicate different regulatory mechanisms for EMPA’s modulation of *AgRP* and *POMC* gene transcriptional regulation.

## 4. Discussion

Obesity and diabetes mellitus are closely related, with weight loss being a crucial factor in managing diabetes. Recent findings suggest that, although SGLT2 inhibitors typically result in weight and blood glucose reduction, some patients may experience increased appetite, leading to greater food consumption [38,39]. A meta-analysis of SGLT2 inhibitors demonstrated a dose-dependent reduction in body weight among patients with type 2 diabetes treated with these agents [40]. Specifically, for EMPA, significant weight reductions of −1.86 kg (95% CI: −2.06 to −1.67) and –1.95 kg (95% CI: −2.11 to −1.78) were observed at doses of 10 mg and 25 mg, respectively [41]. This study investigated the impact of SGLT2 inhibitors on weight and suggested their potential role as therapeutic agents for obesity-related treatment. The hypothalamic neurocircuitry serves as the central regulator of energy balance, with various hypothalamic neuropeptides acting as key modulators within this system. Any malfunction in this highly complex process has been directly linked to the onset of obesity [42,43]. However, the specific molecular mechanisms underlying regulation by SGLT2 inhibitors remain unclear. Therefore, we investigated how SGLT2 inhibitor treatment affects the transcriptional regulation of hypothalamic neuropeptides involved in appetite and energy metabolism. First, we focused on how the hypothalamus responds to changes in appetite and weight. We investigated the transcriptional regulation of hypothalamic neuropeptides involved in the modulation of feeding behavior both in control mice after i.c.v. administration of SGLT2 inhibitors and in HFD-induced obese mice after oral administration of SGLT2 inhibitors. The hypothalamic melanocortin system integrates various peripheral metabolic signals to regulate energy balance. AgRP neurons and POMC neurons coexist in the ARC of the hypothalamus, and they promote or inhibit appetite via the melanocortin pathway [44,45]. In this study, we observed an increase in the expression of *AgRP* and *POMC* transcription at different time-points following central SGLT2 inhibitor treatment. Additionally, using an HFD-induced mouse obesity model to investigate the effects of SGLT2 inhibitors, we found changes in the expression of AgRP and POMC mRNA depending on the duration of SGLT2 inhibitor treatment. Our findings suggest that SGLT2 inhibitors may initially contribute to metabolic regulation mediated by AgRP as a compensatory mechanism, followed by modulation mediated by POMC. However, given the observed increase in food intake over a very long-term period, such as 16 weeks of SGLT2 inhibitor administration, further detailed experiments are needed for future investigations. Additionally, we confirmed that the transcriptional regulation of *AgRP* and *POMC* genes in hypothalamic mHypoA cells is mediated by FoxO1. FoxO1, a transcriptional regulator, is vital for managing energy metabolism and controlling appetite [46,47]. It impacts both AgRP and POMC neurons, which are pivotal in regulating appetite and energy expenditure. In summary, our study confirms that SGLT2 inhibitors regulate two important neuropeptides (AgRP and POMC) in the hypothalamus, indicating that SGLT2 inhibitors may play a significant role in the metabolic regulation mechanism within the hypothalamus. Consequently, the present findings will provide valuable insights into the pharmacological and physiological mechanisms of SGLT2 inhibitor therapy, to develop effective strategies for managing obesity in patients with diabetes.

## 5. Conclusions

In summary, our results suggest that EMPA regulates appetite and energy metabolism-regulating neurons (AgRP and POMC) in the hypothalamus through the transcription factor FoxO1. This regulatory mechanism may play a crucial role in controlling body weight.

## Data Availability

Data are contained within the article.
